# Factors associated with ovine footrot lesions in Uruguayan flocks: a cross-sectional study

**DOI:** 10.3389/fvets.2025.1585564

**Published:** 2025-05-27

**Authors:** Waldemir Santiago Neto, Ana Crescionini, Ludmila Slimovich, Caroline da Silva Silveira, Sofía Salada, Martín Fraga, Sergio Fierro

**Affiliations:** ^1^Plataforma de Investigación en Salud Animal (PSA), Instituto Nacional de Investigación Agropecuaria (INIA), La Estanzuela, Colonia, Uruguay; ^2^Programa de Posgrados, Facultad de Veterinaria, Universidad de la República, Montevideo, Uruguay; ^3^Área de Investigación y Desarrollo, Secretariado Uruguayo de la Lana (SUL), Montevideo, Uruguay

**Keywords:** sheep, *Dichelobacter nodosus*, control strategies, mixed model, multivariable ordinal regression

## Abstract

Ovine footrot has *Dichelobacter nodosus* as the primary pathogen, and it is characterized by its infectious and multifactorial nature, such as environmental conditions, management practices, and host susceptibility, leading to variable prevalence and economic impacts across regions. The present study investigated factors associated with footrot scores in individual sheep from a non-probabilistic sample of 60 flocks enrolled by the Uruguayan Wool Secretariat, from which 6,139 sheep had their feet clinically evaluated from 2021 to 2024. PCR was employed to confirm *D. nodosus* at the farm level, and data on flock management were collected. The occurrence of footrot-related lesions at the animal level was 17.7%, mainly due to severe footrot. Ordinal multivariable mixed models with a random farm effect showed that the intraclass correlation coefficient for farms was 57.2%. Regarding fixed effects, breed stock size, sanitary protocol at sheep admission, formalin footbath, meat production purpose, hoof trimming, and veterinarian assistance for sheep had a protective effect. In contrast, the footrot vaccine and footrot control and eradication program had a risk effect. We conclude that specific management effects influencing *D. nodosus* infection in Uruguayan sheep flocks could guide context-specific, preventive interventions against footrot at the farm level.

## Introduction

*Dichelobacter nodosus* is a Gram-negative, obligatory anaerobic bacterium that is the primary causative agent of ovine footrot, a debilitating infectious disease of the hoof in sheep and other small ruminants (1, 2). It adheres to the interdigital skin and invades tissue, causing inflammation, necrosis, and significant lameness in affected animals, ultimately leading to reduced productivity, welfare concerns, and economic losses in sheep farming systems (1, 3–6). The virulence of *D. nodosus* is driven by its production of extracellular proteases and pili, which facilitate colonization and destruction of host tissues, particularly under warm, moist environmental conditions that promote bacterial survival (7–9). *Fusobacterium necrophorum* plays a secondary role in lesion progression. Traditionally considered an environmental contaminant, recent findings suggest that *F. necrophorum* persists within the sheep interdigital skin rather than solely in the environment, contributing to the chronicity and recurrence of footrot (10).

The epidemiology of footrot is complex, influenced by the virulence of the bacteria, environment, host susceptibility, and management factors. Warm and wet climates favor the survival and spread of *D. nodosus*, leading to higher disease prevalence in these regions. Globally, footrot remains a significant health issue, with an estimated prevalence ranging from 16 to 42% in affected flocks, depending on the geographic region, season, and control measures implemented (11–13). The disease is transmitted through direct contact between infected and susceptible animals or via contaminated pastures, with carriers playing a critical role in maintaining the infection within flocks (14).

Control measures for footrot include a combination of vaccination, regular foot inspections, targeted treatment, biosecurity, and environmental management. For instance, vaccination campaigns in Australia, Bhutan, and Nepal, using mono- or bivalent vaccines, have significantly reduced footrot prevalence (15–17). In the United Kingdom, the adoption of the Five-Point Plan, which emphasizes prompt treatment, quarantine, and culling of chronic cases, has demonstrated a marked reduction in disease burden (2). In some Scandinavian countries, eradication programs focusing on strict culling and biosecurity have successfully eliminated footrot from national sheep populations (18, 19).

In Uruguay, footrot has been recognized as an endemic challenge for sheep farming, particularly because of the favorable environmental conditions of the country. However, current knowledge regarding the epidemiology of the disease in the country remains limited. A previous study in Uruguay carried out in 1999 utilized a two-stage random sampling method involving 153 farms and 13,357 sheep (20). The sampling methodology included selecting farms proportional to their flock size and inspecting animals from each category (rams, ewes, lambs) for clinical signs of footrot. Lesions were classified using a modified (21) scale from 0 to 5 (22), which includes a virulence marker (elastase activity) to the clinical signs for the scoring basis, and laboratory testing confirmed the presence of *D. nodosus* through immunofluorescence. There was a prevalence of 6.6% among the sheep population and 69.7% at the farm level, indicating a widespread distribution of footrot across the country. The highest prevalence was found in rams (19.8%), followed by breeding ewes (7.4%) and lambs (3.9%). Risk factors included environmental conditions such as humidity and soil type, although there were no strong statistical associations with specific management practices like paddock size or forage improvement. However, such matters as the inadequate use of footbaths and low rates of veterinary assistance were noted as areas for improvement. Regarding production parameters, Mederos (20) found that severe footrot significantly and negatively affects live weight and body condition. Sick animals were 3.8% lighter than healthy animals, reaching 9.7% in times conducive to the disease. Regarding wool production, a decrease in fiber yield and strength was observed.

Whilst footrot outbreaks have been extensively studied in developed countries, there is a lack of evidence regarding associated factors in developing countries. Non-probabilistic cross-sectional studies, while limited in their ability to provide representative population estimates, offer valuable insights into disease dynamics, especially in resource-constrained settings. Here we address this gap by describing and analyzing footrot outbreaks in Uruguayan sheep flocks covering a 3-year survey. A study sampling most of the departments in Uruguay provides a useful overview of footrot occurrence and its associated risk factors under specific environmental conditions. Such studies are particularly beneficial for generating hypotheses, identifying trends, and informing targeted control strategies in the absence of probabilistic sampling frameworks (23). This paper aims to contribute to the knowledge of the epidemiology of footrot in Uruguayan ovine production systems. The objective of this study was to identify the associated factors with footrot lesions in most of the Uruguayan regions.

## Materials and methods

### Study area and target population

Uruguay has a total area of 176,215 km^2^, 19 departments, and 112 municipalities and shares borders with Argentina and Brazil. The sheep population was approximately 5.9 million in 2023, mainly distributed in the north of the country. According to the official data from the Ministry of Livestock, Agriculture and Fishery of Uruguay (MGAP), 21,434 flocks were registered in 2023 (24). The target population of this study included sheep flocks affected by footrot outbreaks and footrot-free flocks.

**Figure 1:**
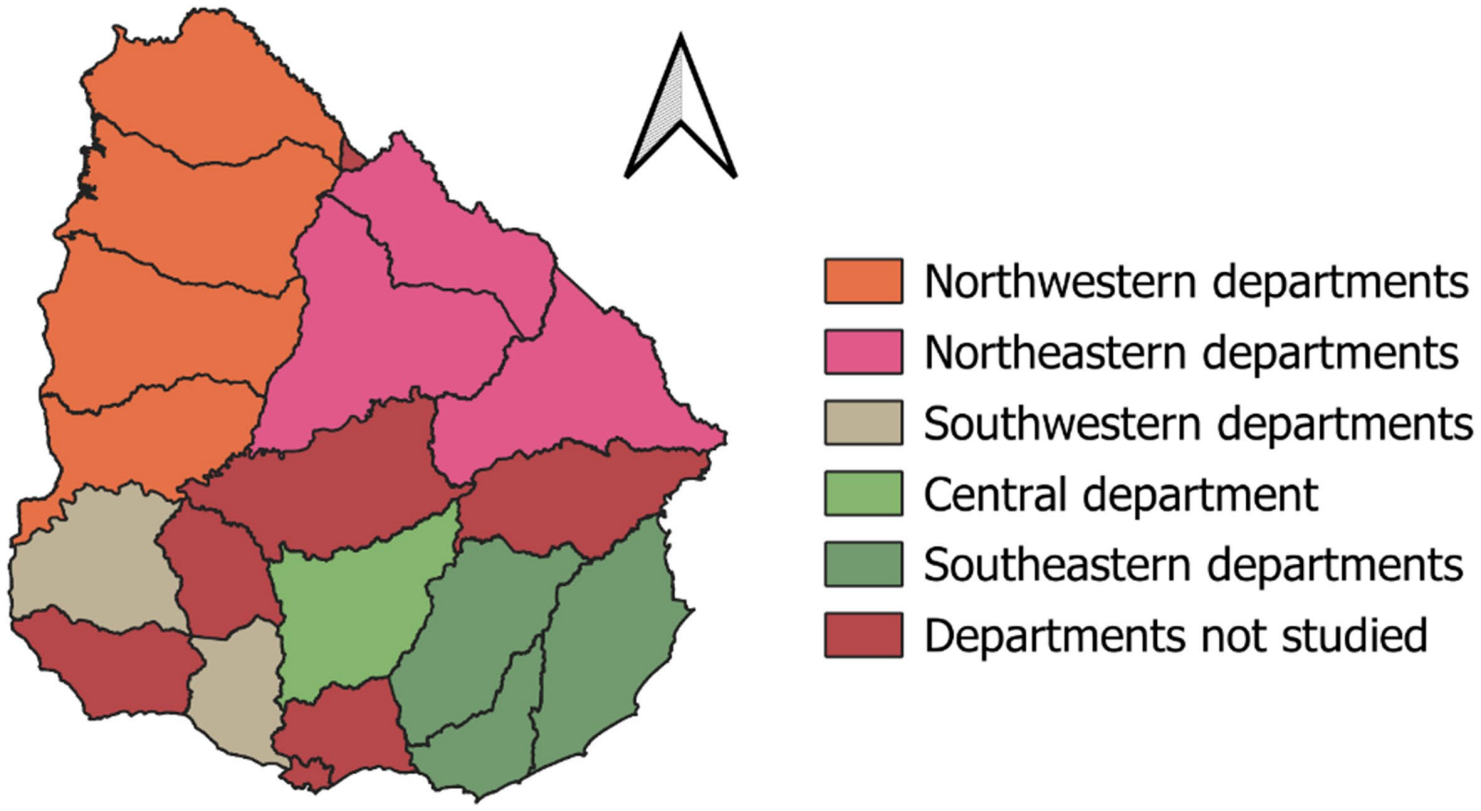
Spatial distribution of the 60 studied sheep flocks across departments in Uruguay, surveyed between 2021 and 2024.

The studied farms were distributed all over the country, including northwestern departments Artigas, Salto, Paysandú, Río Negro; northeastern departments Rivera, Tacuarembó, and Cerro Largo; one central department Florida; Southwestern departments Soriano and San José; and Southeastern departments Lavalleja, Rocha and Maldonado (Figure 1). The period from January 2021 to January 2024 was marked by higher temperatures, reduced humidity in drought-affected areas, and extreme precipitation deficits. The northern and central regions experienced notable heat stress, while the coastal areas remained relatively milder but still suffered from prolonged dry conditions. The 2022 drought was severe in much of the country, significantly impacting agriculture and water availability (25).

The research was approved by the Ethics Commission for the Use of Experimental Animals of INIA (CEUA), registered with the CNEA under registration number 0009/11.

### Survey design, sample collection, and disease severity scoring

We aimed to identify factors associated with the *D. nodosus*-related lesions in sheep flocks. During the cross-sectional study, Uruguay had a drought, which possibly masked, to some degree, the disease. In this context, from January 2021 to January 2024, we conventionally sampled 60 flocks, which either contacted the Uruguayan Wool Secretariat or were indicated to participate in the study by third parties. The sample included footrot-free flocks and those experiencing problems with the disease recently or repeatedly, and all recruited farmers participated voluntarily. The veterinarians of the study performed clinical examinations for up to 100 animals per flock, assessing the lesions on each hoof using a scoring system from 0 to 4 (Table 1) (21). This system was used instead of the standard 0–5 system once the elastase virulence marker was not considered [as in Mederos (20) and Stewart (22)]. We clinically examined 6,139 sheep and sampled lame-affected sheep, sampling the foot with the highest score of the lesions in case more than one foot was affected. Flock sizes ranged from 100 to 7,376, with a mean sample size per flock of 102.3 sheep. The number of flocks studied ranged from 1 (in Río Negro and San José departments) to 9 (in Salto department), while the number of clinically inspected sheep varied from 47 (in Lavalleja) to 1,940 (in Salto) (Table 2). On each farm, located in one of the thirteen Uruguayan departments, the veterinarians interviewed the sheep farmer during the visit on the sampling day and documented the answers on a written questionnaire (Supplementary material).

**Table 1:** Three-level score classification for ordinal regression based on the scoring system from (21).

Analysis score	Lesion assessment score	Lesion description
0	Score 0	Normal feet
1	Score 1	Mild interdigital dermatitis
	Score 2	Severe interdigital dermatitis
2	Score 3	Severe interdigital dermatitisand under-running of the horn of the heel and sole
	Score 4	As 3 but with the underrunning extended to the walls of the hoof

For the confirmation of *D. nodosus* infection at the flock level, samples of the lesions were taken using sterile swabs for each injured interdigital space and the hoof of 22 affected animals per flock, representative of the mobs (e.g., logistically separated sheep groups in the farms), categories (e.g., ewes, lambs, rams), and degrees of lesions (from mild to severe) (26) with prior removal of the gross dirt by wiping one thumb through the interdigital space without touching the interdigital skin (27). When there were typical lesions, the sample was taken from the active area. If there was more than one affected foot of a single sheep, the foot with the highest score was sampled for posterior analysis. Latex gloves were changed between sampled animals to avoid cross-contamination. Once the sample was collected, the swab was stored in microtubes containing 800 μL of DNA/RNA Shield (ZYMO) at room temperature and arrived at the laboratory approximately 48 h after collection. Clinical footrot cases were confirmed by PCR.

**Table 2:** Distribution of the number of flocks studied and the number of animals clinically inspected in each department.

Region	Department	Number of flocks	Number of animals inspected
Northwestern	Artigas	5	500
	Salto	9	1940
	Paysandú	7	700
	Río Negro	1	100
Northeastern	Rivera	6	600
	Tacuarembó	7	661
	Cerro Largo	5	428
Central	Florida	3	180
Southwestern	Soriano	6	534
	San José	1	73
Southeastern	Lavalleja	2	47
	Rocha	5	210
	Maldonado	3	166

### Sample processing/laboratory analysis

The samples were processed in the Microbiology Laboratory of the La Estanzuela Animal Health Platform of the Instituto Nacional de Investigación Agropecuaria. *D. nodosus* was detected using the PCR technique (28). DNA extraction from the swabs sent to the laboratory was performed according to (29).

### Questionnaire and interview

A questionnaire regarding putative factors associated with footrot within a flock (Supplementary material) was applied during visits to 53 (who agreed to participate) out of the 60 selected sheep farmers (88.3%). The structured questionnaire was developed by advisory from the veterinarians of the survey and based on previous studies, containing 40 “closed-ended” questions grouped into five main categories: general farm characteristics (including breeds reared, e.g., Australian Merino, Crossbreed for meat purposes, Texel, Dorper, Corriedale, Corriedale Pro, Highlander, Ideal, Dohne Merino, Merílin, Milchschaf, and Romney Marsh), biosecurity (including reproductive management), farm sanitary conditions, general management and farm facility structure, and specific disease control. The questionnaire was tested with three non-participating farmers to identify potential sources of misinterpretation and then refine the questions accordingly. Personal interviews (face-to-face) lasted between 20 to 40 min and were conducted by four trained veterinarian interviewers.

### Statistical analysis

All collected variables were tested for frequency distribution; continuous variables were tested by histogram, mean, standard deviation, and range and categorized according to quartiles. The statistical process was carried out with R-language v.4.2.2 (‘ordinal’ package; R Development Core Team, 2022). Variables with large amounts of missing data (> 10%) and limited variability (< 20%) were not included in the univariable analysis.

#### Ordinal logistic regression

Stepwise variable selection was used to select the most relevant predictors from a set of candidate variables in an ordinal logistic regression, e.g., foot health management practices on footrot. The dependent variable was defined in categories of the footrot severity into ordinal levels: 0 = No footrot; 1 = Mild footrot; 2 = Severe footrot. Candidate predictor variables included, for example, hoof trimming, antibiotic treatments, pasture rotation, hygiene measures, vaccination status, and environmental conditions (e.g., rainfall).

We ensured that there was no multicollinearity among predictors (using correlation matrices and variance inflation factor [VIF]). We fitted an initial ordinal logistic regression model with a random intercept (e.g., proportional odds model) using all candidate predictors:


$$\log\frac{P(Y_{\mathrm{ij}}\leq k)\;}{P(Y_{\mathrm{ij}}>k)}=\beta_{0}^{\left(k\right)}+X_{\mathrm{ij}}^{T}\beta +u_{j}\;$$

Where $Y_{\mathrm{ij}}$ = ordinal outcome (e.g., footrot severity) for the *i*-th sheep in the *j*-th flock; k = the category threshold (e.g., 0 = none, 1 = mild, 2 = severe); $\beta_{0}^{\left(k\right)}\;$ = threshold-specific intercepts (cut-points); $X_{\mathrm{ij}}\;$ = vector of predictor variables for sheep *i* in flock *j*; β = vector of fixed-effect coefficients; $u_{j}$ ~ $N(0,\sigma_{u}^{2})\;$ = random intercept for flock *j*, capturing flock-level deviation.

We performed stepwise variable selection using statistical criteria such as the Akaike Information Criterion (AIC), proceeding in a forward selection. First, beginning with no predictors in the model, followed by adding predictors one at a time based on the greatest improvement in model fit (e.g., reduction in AIC), and stopping when no additional predictor significantly improves the model. Confounding effects were investigated by checking changes in the point estimates of the variables that remained in the model. Variables that change parameter estimates > 25% were considered confounders and retained in the model. Finally, two-way interaction terms between variables with biological plausibility were investigated. After selecting the final set of predictors, the model goodness-of-fit was assessed through McFadden’s pseudo R^2^.

## Results

**Figure 2:**
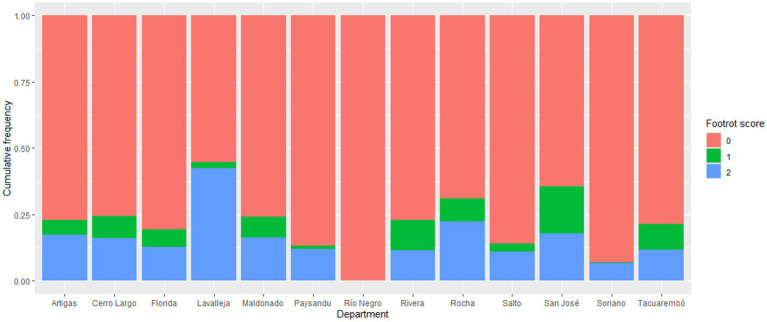
Distribution of the categories of footrot lesions in the inspected sheep in each studied Uruguayan department. 0 = absence of lesions; 1 = mild lesion (less than score three on the grade scale); 2 = severe lesions (equal or higher than score three on the grade scale).

This study aimed to determine which variables would be associated factors with footrot lesions in a conventional sample of flocks from most of the departments of Uruguay. There were 1,088 footrot-affected sheep from 55 out of 60 flocks, resulting in an estimated occurrence at the animal level of 17.7% (CI_95%_ = 16.8–18.7), including all scores. Of those, 766 (12.5%; CI_95%_ = 11.7–13.3) and 322 (5.2%; CI_95%_ = 4.7–5.8) sheep had severe and mild footrot, respectively. Furthermore, we did not identify sheep positive for *D. nodosus* without lesions. The Lavalleja department in the southeastern region showed the highest percentage of severe lesions (Figure 2), twice the study’s value, due to important outbreaks in small flocks, typical in this region. In the same area, the Rocha department had the second highest percentage of severe footrot. Conversely, the departments of Río Negro and Soriano showed the highest rate of footrot absence, likely due to the enrollment of control flocks from these regions. The cumulative frequency of positive samples by each department can be observed in Figure 2.

Three independent variables showed a VIF > 2.5: breed stock size (ewes), total stock size, and specific area for sheep rearing. ´Breed stock size´ was kept in the model because it had the lowest *p*-value. In the univariate analysis, 21 variables presented a *p*-value ≤ 0.20 (Table 3) for the presence of footrot. The final model identified eight variables as significantly associated with footrot (*p* < 0.05) (Table 4): the implementation of a sanitary protocol at sheep admission (OR = 0.07; CI_95%_ = 0.03–0.13; *p* < 0.001), the formalin footbath (OR = 0.22; CI_95%_ = 0.13–0.39; *p* = <0.001), the footrot vaccination (OR = 4.5; CI_95%_ = 2.3–9.2; *p* = <0.001), the implementation of a footrot control and eradication program (OR = 6.7; CI_95%_ = 3.9–11.3; *p* < 0.001), the rearing for meat production purpose (OR = 0.5; CI_95%_ = 0.3–0.8; *p* = 0.003), hoof trimming (OR = 0.4; CI_95%_ = 0.2–0.7; *p* = 0.003), and the veterinarian assistance for sheep (OR = 0.5; CI_95%_ = 0.3–0.8; *p* = 0.003). None of the two-way interaction terms were significant at a 5% significance level, and the breed stock size was the only confounding effect forced into the model once it changed the parameter estimates by more than 25%. McFadden’s pseudo-R^2^ was 44.6. The intraclass correlation coefficient for farms was 57.2.

**Table 3:** Definition and distribution of explanatory variables retained at the univariate analysis*.

Variables	No of animals	Frequency (%) or median	*p*-value	OR (95% CI)
Veterinarian assistance for sheep	4,546		0.12	
No		36		–
Yes		64		0.8 (0.6–1.1)
Rams bought	4,546		0.16	
No		21		–
Yes		79		1.4 (0.9–2.2)
Meat purpose	4,740		0.01	
No		64		–
Yes		36		1.5 (1.1–2.2)
Footbaths in good condition	3,793		<0.001	
No		26		–
Yes		74		0.4 (0.3–0.6)
Specific area for sheep rearing^a^	4,546	1105	0.002	1.0002 (1.000004–1.0004)
Footrot control and eradication program	4,546		0.001	
No		49		–
Yes		51		1.7 (1.2–2.4)
Veterinarian in charge of the control and eradication program	2,336		0.0002	
No		26		–
Yes		74		4.1 (1.9–8.5)
Footrot diagnosis by the producer/owner	4,446		<0.001	
No		47		–
Yes		53		0.3 (0.2–0.4)
Footrot diagnosis by the veterinarian	4,546		<0.001	
No		63		–
Yes		37		3.7 (2.3–5.7)
Formalin footbath	4225		<0.001	
No		50		–
Yes		50		0.5 (0.4–0.7)
Fair-like events participation	4,546		<0.001	
No		70		–
Yes		30		0.3 (0.2–0.5)
Breed stock size^a^	4,546	729.6		
Below 340		37.9		–
Between 341 and 720		22.5	0.34	0.8 (0.5-1.2)
Between 721 and 1300		22	0.04	1.7 (1-2.8)
Above 1300		17.6	<0.001	3.7 (2.1-6.5)
Improved pasture feeding	4,546			
No		39		–
Yes		61	0.03	1.5 (1–2.1)
Rainfall (mm)	4,246	1050.9	<0.001	1.001 (1.0006–1.0014)
Total stock size^a^	4,446	1918.9	0.002	1.0001 (1.00002–1.00018)
Footrot vaccine	4,546		<0.001	
No		70		–
Yes		30		1.9 (1.3–2.6)
Breed	4,740			
Australian Merino		36		–
Cross breed for meat purposes		9	0.07	2.4 (0.9–6.2)
Corriedale		29	<0.001	0.3 (0.16–0.53)
Highlander		6	0.3	0.6 (0.2–1.5)
Ideal		4	0.01	0.07 (0.01–0.5)
Dohne Merino		14	0.06	0.66 (0.42–1.01)
Merílin		2	<0.001	0.12 (0.04–0.4)
Sharing sheep dipping	4,546			
No		69	0.001	–
Yes		31		2.2 (1.4–3.7)
Sanitary protocol at sheep admission	4,369		<0.001	
No		76		–
Yes		24		0.37 (0.24–0.57)
Meat production purpose	4,740		0.03	
No		64		–
Yes		36		1.7 (1.2–2.3)
Hoof trimming	4,226		0.004	
No		60		–
Yes		40		0.56 (0.38–0.83)

a Variable used to control potential confounding, with categories defined according to quartiles. * Cumulative link mixed model with logit link function.

**Table 4:** Final ordinal logistic regression mixed model with the variables significantly associated with footrot (*n* = 3,615 samples).

Variable	Estimate (*β*)^a^	S.E.^b^	*p*-value	OR (95% CI)
Confounder (Breed stock size)^c^				
Below 340	Ref.	–	–	–
Between 341 and 720	−1.6	0.3	<0.001	0.2 (0.1–0.3)
Between 721 and 1,300	−1.5	0.4	<0.001	0.2 (0.1–0.5)
Above 1,300	−0.8	0.5	0.07	0.4 (0.2–1.1)
Associated factors				
Sanitary protocol at sheep admission				
Yes	−1.6	0.3	<0.001	0.07 (0.03–0.13)
No	Ref.	−		−
Formalin footbath				
Yes	−1.5	0.3	<0.001	0.22 (0.13–0.39)
No	Ref.	–		–
Footrot vaccine				
Yes	1.5	0.4	<0.001	4.5 (2.2–9.2)
No	Ref.	–		–
Footrot control and eradication program				
Yes	1.9	0.3	<0.001	6.7 (3.9–11.3)
No	Ref.	–		–
Meat production purpose				
Yes	−0.7	0.2	0.003	0.5 (0.3–0.8)
No	Ref.	–		–
Hoof trimming				
Yes	−0.9	0.3	0.003	0.4 (0.2–0.7)
No	Ref.	–		–
Veterinarian assistance for sheep				
Yes	−0.7	0.2	0.003	0.5 (0.3–0.8)
No	Ref.	–		–

^a,b^Results are given with Estimate (β), standard errors (S.E.), *p*-values, and OR with 95% CI. ^c^Categories were defined according to quartiles.

## Discussion

We identified six protective practices and two positively associated risk factors for footrot lesions. The strongest protective association came from a sanitary protocol implementation at sheep admission to the flock, a strategy that has been previously validated for its efficacy in controlling infectious disease in small ruminants (7, 30). The sheep admission protocol encompasses seven critical points: prior knowledge of the sanitary status of the origin flock (including its production and reproduction indices and vaccination history for diseases like contagious ecthyma and clostridial infections), mob-level and individual clinical inspections of incoming sheep, control of endo- and ectoparasites, foot conditions assessments with segregation of chronic carriers and treatment of healthy or mildly affected sheep using footbaths (31, 32). The latter measures are particularly effective in minimizing the introduction of *D. nodosus* and managing subclinical carriers (33).

Breed stock size (number of ewes) and formalin footbath showed the second-highest protective effects. For breed stock size, intermediate low and high sizes were associated with a protective effect, and the highest breed stock sizes demonstrated a tendency towards protection. It likely reflects the challenges small flocks face in managing outbreaks due to resource constraints, as there was a strong correlation (r = 0.96) between breed (ewes) and total stock size. Larger flocks, often better resourced, can implement more consistent biosecurity practices, preventing outbreaks. Small flocks, however, may experience higher labor demands when affected, which limits their ability to rear larger numbers of sheep with footrot. These findings align with prior reports highlighting the relationship between flock size, management capacity, and disease prevalence (34).

Footbath practices, particularly the use of formalin, have been extensively studied. The results from the multivariable model suggest a positive effect of formalin footbath on footrot control, considering general adequate footbath structure condition (e.g., self-reported in 76% of the farms) and no report or inspection of granulomas – a common complication due to formalin misapplication (35). Despite their toxic effect (the reason it is banned from some countries), if properly applied in frequency and concentration, it seems beneficial. Thus, there is a need for precise adherence to recommended concentrations and protocols to maximize benefits while minimizing adverse effects (35). Similarly, there was mixed farmer satisfaction with current footbath practices, noting that while effective when applied correctly, farmers often struggle with the practicalities of consistent application (36). It also emphasized the importance of combining footbaths with other preventive measures to achieve comprehensive footrot management (36).

Furthermore, it was demonstrated that integrating flock-specific lameness control plans, including tailored footbath protocols, significantly reduces the prevalence of footrot in sheep flocks (37). Their findings underline the importance of adapting practices to the unique conditions of each flock while providing structured guidelines (37). Kaler and Green (38) note that while footbathing is widely adopted, its efficacy depends on consistent implementation alongside other management practices, such as regular inspection and prompt treatment of affected animals (38). Their research reinforces the need for holistic approaches to lameness control (38). A nuanced perspective on the role of footbaths, advocating for precision, consistency, and integration with broader management strategies, seems to be the best approach (34).

Following protective associated factors, there was hoof trimming. Once considered a cornerstone of lameness management in sheep, it has become increasingly controversial due to emerging evidence of its potential to harm rather than benefit animal health. A strong association between routine hoof trimming and the development of granulomas and shelly hoof has been identified, suggesting that excessive or inappropriate trimming can cause microtrauma, creating entry points for pathogens and exacerbating lameness (35). Similarly, Green et al. (39) argue that evolving “best practices” now recommend minimal or targeted trimming only when necessary, contrasting with traditional views that favored regular trimming as a proactive measure. As we assessed the frequency of hoof trimming as an open question, it was difficult to systematically analyze if the protective association of this practice was due to regular and proactive (e.g., in the summer, every year) or minimal and targeted hoof trimming (e.g., sporadically or when deemed necessary).

Nevertheless, while there was no association between footbath use and hoof trimming, as hypothesized, there was a significant association between good footbath condition and hoof trimming (data not shown) – which did not persist in the multivariate model. It suggests a general focus on foot health care. This shift highlights the need for farmers to adapt to new evidence, even if it conflicts with long-standing practices (39). Further complicating the adoption of new trimming guidelines is the gap between theoretical “best practices” and on-farm realities. While agricultural educators advocate for modern approaches, both practical constraints and ingrained habits among farmers often result in outdated methods persisting in the field (10). This misalignment underscores the need for improved communication and tailored training to facilitate change (10). Farmers who embraced evidence-based lameness control plans, which include minimizing unnecessary hoof trimming, achieved lower lameness prevalences (2). Combining trimming moderation with holistic management strategies, such as those outlined in the Five-Point Plan, is key to reducing lameness effectively (2). They emphasize the need for a paradigm shift in hoof-trimming practices grounded in current research and supported by education and resources for practical implementation.

The least protective among the protective variables were both the regular assistance from a veterinarian in the rearing process and the rearing of sheep for meat production. Veterinarians’ assistance plays a pivotal role in disease control on sheep farms. Yet, its impact is heavily influenced by farmer attitudes, perceptions, and relationships with veterinary professionals. Historically, in Uruguay, most sheep farmers did not have veterinary assistance (20). Farmers prioritizing animal welfare are more likely to engage with veterinarians and adopt recommended practices, emphasizing the importance of aligning veterinary advice with farmer values (40). Similarly, farmer beliefs, emotions, and perceived barriers, such as cost or skepticism towards veterinary recommendations, can limit the uptake of evidence-based practices like those for footrot prevention (41). Veterinarians fostering collaborative and trust-based relationships with farmers support effective flock health management (42). In Spain, veterinary services were statistically significantly involved in reducing brucellosis, emphasizing that farms with stronger veterinary engagement showed lower disease prevalence (43). Personalized, one-to-one veterinary interventions, combined with tailored message framing, significantly improve the adoption of management practices for conditions like lameness (44). Veterinarians play a critical role in sheep farming by delivering high-quality, cost-effective advice to secure their position within the industry. The challenges veterinarians face – competition from non-professional advisors and the need to demonstrate value to farmers – suggest that proactive engagement and specialized knowledge are essential for veterinarians to remain integral to sheep health management (45). Participatory veterinary services demonstrate that when farmers actively participate in disease management strategies, veterinary service delivery becomes more effective and impactful (23). The multifaceted role of veterinarians in disease control, combining technical expertise with a tailored farmer-centered approach, maximizes their influence.

Breed purpose appeared to influence susceptibility to footrot, with meat-related breeds such as Texel, Dorper, and Highlander, and dual-purpose breeds like Corriedale, Corriedale Pro, Ideal, Romney Marsh, and Milchschaf showing greater resilience compared to wool breeds such as Merino. These findings are consistent with previous research that identified breed-related susceptibility as a significant risk factor for *D. nodosus* infection (46). That study revealed marked genetic and phenotypic differences in footrot susceptibility among breeds, highlighting traits such as denser wool and smaller hooves – common in hill breeds like Merino – may predispose sheep to higher disease risk due to increased environmental exposure and hoof conditions favorable to bacterial colonization. In contrast, lowland and meat-focused breeds with larger hooves or those selected for resistance, such as Ile-de-France and Suffolk, were associated with lower prevalence and milder disease outcomes (46). These insights support the integration of breed-specific strategies and genetic selection into footrot control programs. Complementarily, Bhardwaj et al. (47) examined conformation traits in Merino sheep and found that although variables such as lateral heel height, interdigital skin height, and sole width were significantly associated with lesion severity in univariable analyses, they did not retain significance in multivariable models – underscoring the need for larger, more detailed studies to clarify the phenotypic basis of breed susceptibility.

Due to the cross-sectional nature of this work, it was not possible to determine whether footrot vaccination was implemented as a preventive measure or in response to an outbreak, limiting the ability to assess its effectiveness within the sampled flocks. There is one multivalent commercial vaccine available in Uruguay, which contains the serogroups B, C, D, E, F, G, and H. Although we found 9 out of 10 known serogroups, most flocks did not present more than two serogroups (i.e., 70%; data not shown). It was demonstrated that multivalent vaccines suffer from immunological competition, which results in reduced antibody production against each vaccine component (48). The use of footrot vaccines in sheep flocks remains a debated component of lameness management strategies, with its effectiveness influenced by factors such as timing, administration protocols, and disease context. While vaccination can play a role in a comprehensive lameness control strategy, its impact is maximized when integrated with evidence-based practices, such as prompt treatment and environmental management, as outlined in the Five-Point Plan (2). However, reliance on vaccination alone, without concurrent use of other evidence-based methods, may lead to suboptimal outcomes (14). Reductions in prompt treatment practices were associated with increased lameness prevalence, suggesting that vaccination should complement rather than replace other management measures (14).

While vaccine use has been associated with reduced lameness prevalence in some flocks, its efficacy is often contingent on farm-specific factors, such as the presence of a highly virulent strain of *D. nodosus* or the timing of administration relative to outbreaks, contrasted with the vaccine formulation (15, 49), which could explain the positive association in our study. Moreover, the availability of only multivalent vaccines in certain regions (e.g., as in Uruguay) may limit their effectiveness due to immunological competition, highlighting the need for region-specific solutions and ongoing refinement of vaccine formulations (49). In contrast to multivalent vaccines, targeted monovalent vaccines have shown greater success in eradicating specific *D. nodosus* strains, suggesting that region-specific vaccine strategies could enhance control efforts (15). Vaccination is highlighted as part of an integrated approach, combining it with the culling of chronic carriers, improved biosecurity, and strategic antibiotic use to maximize long-term disease eradication efforts (50). It underscored the need for continuous refinement of vaccination protocols to adapt to evolving epidemiological challenges in different sheep-rearing regions (50).

Finally, a footrot control and eradication program showed the highest odds ratio, possibly indicating either a chronic, long-lasting footrot problem at the farms or a recent intent to combat the disease. Lameness control programs, particularly those targeting footrot, benefit significantly from a tailored, flock-specific approach (37). Their study, conducted through a stepped-wedge trial on 44 English sheep flocks, highlighted that bespoke management plans addressing unique conditions and challenges of individual flocks achieved measurable reductions in lameness prevalence. Key components of these plans included regular monitoring, prompt treatment of affected sheep, and preventive measures such as footbathing and vaccination. The study emphasized the role of farmer engagement and education in the success of such programs, showing that involving farmers in decision-making and providing practical guidance increased compliance and effectiveness. These findings underscore that one-size-fits-all approaches are less effective than strategies considering specific flock dynamics, environmental conditions, and resource availability (37). Although there is a plan tailored to Uruguayan conditions, it may not be fully implemented, or it may be done with errors in diagnosis or treatment, exemplified by the high percentage of flocks that did not have a veterinarian diagnosing footrot (e.g., 63%; Table 3).

One of the cornerstones of the program is the culling of irrecoverable footrot-affected sheep, usually in the summer. Culling persistent lame sheep can be an effective measure within a lameness control program, particularly for managing chronic cases of footrot (37). By removing individuals who do not respond to treatment or are recurrently affected, farmers can reduce the overall burden of infection in the flock, thereby minimizing the risk of disease transmission and improving the efficacy of other control measures, such as prompt treatment and preventive practices. However, the study also highlighted the importance of using culling as a complementary strategy rather than a standalone measure, as its success depends on robust flock monitoring, early intervention, and the implementation of a comprehensive management plan (37). Hence, the positive association between footrot and an ongoing control program could reflect a lack of compliance with the control program’s standards.

An intraclass correlation coefficient (ICC) of 57.2% indicates that a substantial proportion of the variability in footrot occurrence was attributable to differences between farms rather than the evaluated factors. This high ICC suggests that farm-level characteristics – such as environmental conditions or regional climate – shape footrot occurrence, potentially overshadowing the assessed management practices. The ICC value implies that farms with similar conditions exhibit comparable footrot occurrence, reinforcing the need for farm-specific interventions rather than generic, one-size-fits-all recommendations. Additionally, the magnitude of this ICC underscores the importance of considering farm-level random effects in statistical models to accurately capture clustering effects and avoid overestimating the influence of specific management practices.

We acknowledge some limitations. This study’s inference capacity is constrained by its reliance on non-probabilistic sampling, which limits the generalizability of the findings to broader populations. Additionally, the cross-sectional design captures data at a single point, providing a snapshot rather than a dynamic understanding of disease trends. The study was conducted during a drought, a condition unfavorable for *D. nodosus* spread that thrives in warm and wet environments. Consequently, the occurrence and associated factors identified may not fully represent typical patterns observed under conditions conducive to the bacterium’s expression. These limitations highlight the need for caution when extrapolating results to other settings or environmental contexts, as well as the importance of longitudinal studies conducted across diverse weather and landscape conditions to obtain a more comprehensive understanding of the factors influencing *D. nodosus* occurrence and transmission. However, the three-year research period and broad territorial sampling coverage, the high response rate of participants to the epidemiological questionnaire, and the mixed modeling with the control of multicollinearity of the variables sought to mitigate potential biases in the study.

## Conclusion

This study, conducted during a drought period across conventionally selected Uruguayan sheep flocks, identified six protective factors and two risk factors associated with ovine footrot. Larger flock sizes and the rearing of meat-purpose breeds, linked to lower odds of disease, suggest structural and genetic influences on footrot resilience. Among footrot-specific management practices, the formalin footbaths and regular hoof trimming showed protective effects, reinforcing their value within integrated control strategies. Additionally, biosecurity measures associated with reduced footrot occurrence, such as implementing a sanitary protocol at sheep admission and maintaining veterinary assistance, underscore the importance of preventive health frameworks. Conversely, footrot vaccines and control and eradication programs associated with higher odds of disease likely reflect their application in response to existing or severe outbreaks (e.g., multiple-serovar or highly virulent outbreaks) rather than ineffectiveness. These findings can inform evidence-based interventions by supporting tailored strategies that prioritize biosecurity, flock structure, and preventive management practices while encouraging critical evaluation of the timing and context in which vaccines and eradication programs are introduced.

## Data Availability

The raw data supporting the conclusions of this article will be made available by the authors, without undue reservation.
