# Supplementary material for: Factors associated with ovine footrot lesions in Uruguayan flocks: a cross-sectional study
Source: Front Vet Sci. 2025 May 27;12:1585564. doi: 10.3389/fvets.2025.1585564 (PMC12148904; doi:10.3389/fvets.2025.1585564)
Supplement: Supplementary file 1 [file Data_Sheet_1.PDF]

Estudio epidemiológico de Footrot (pietín) en Uruguay

Fecha: _________

Nombre de la propiedad: ____

Propietario: ____

Departamento: ____

Ubicación del predio: ____

DICOSE: ____

Propiedad es: CASO ( ) CONTROL ( )

Características generales del predio:

1. Superficie total: Hectáreas dedicadas a ovinos:
2. Tipo de suelo:
3. Pastoreo ovino: Campo natural ( ) Mejoramientos ( )
   a. Cuales mejoramientos:
   b. N° hectáreas:
4. Condición de alambrados perimetrales: Buena ( ) Regular ( ) Mala ( )
5. Precipitación aproximada anual (mm):
6. a. Cuantas personas trabajan en el establecimiento? ___________ b. Cuantas dedicadas a ovinos? _____________ c. ¿Hace cuánto cría ovinos? ________ d. ¿Hay mucha rotación de personal que trabaja con los ovinos? Cuantificar (ej. Tiempo promedio de cambios)
7. Raza:
8. a. Objetivo de la producción: _____________________ b. ¿Posee certificación? Responsible Wool Standard ( ) Ecológica ( ) Otra: __________
9. Número de ovinos en el predio
   a. Stock total:
   b. Majada de cría:
10. ¿Crían ganado vacuno? SI ( ) NO ( )
11. ¿Realiza pastoreo mixto? SI ( ) NO ( )
12. ¿En qué momento realiza la encarnerada?
    a. Realiza: ( ) Inseminación ( ) Monta natural ( ) Monta dirigida
13. ¿En qué momento realiza la esquila?
14. ¿Suplementa alguna categoría? SI ( ) NO ( )
    a. ¿Qué categoría? Ovejas de cría ( ) Ovejas descarte ( ) Borregas 2 – 4 D ( ) Corderas ( ) Corderos ( ) Borregos ( ) Capones ( ) Carneros ( )

b. ¿En que momento?

15. ¿En algún momento del año realiza encierros en el cual los animales permanezcan a alta carga? SI ( ) NO ( )
    a. ¿Qué categoría? Ovejas de cría ( ) Ovejas descarte ( ) Borregas 2 – 4 D ( ) Corderas ( ) Corderos ( ) Borregos ( ) Capones ( ) Carneros ( )
    b. ¿En qué momento?

16. ¿Posee asistencia veterinaria dedicada a ovinos en el predio? SI ( ) NO ( )
    a. Anual ( ) Semianual ( ) Mensual ( ) Solo cuando se solicita ( )
    b. ¿Crees que hay asistencia veterinaria disponible para manejar el pietín en tu región? SI ( ) NO ( )
    c. ¿En cuál grado de facilidad es el acceso del veterinario?
       Sencillo ( ) Moderado ( ) Difícil ( )

Bioseguridad y condiciones sanitarias:

17. a. Hay cuantas propiedades vecinas? ______ b. ¿Hay cuantas propiedades vecinas que producen ovinos? ______
18. ¿Lleva animales a eventos de aglomeración?
    SI ( ) NO ( ) Si sí, donde? Exposiciones ( ) Remates ( ) Ferias ( )
19. ¿Los carneros son producidos en el predio o compra fuera?
    a. En el caso de comprar fuera, ¿tiene en cuenta que la cabaña sea libre de pietín?
20. ¿Utilizan carneros compartidos con otros productores? SI ( ) NO ( )
    a. ¿Observó lesiones podales en el/los mismo/s? SI ( ) NO ( )
21. a. ¿Posee baño de inmersión? SI ( ) NO ( )
    b. Si sí ¿lo comparte con vecinos? SI ( ) NO ( )
22. ¿Introduce animales al establecimiento? SI ( ) NO ( )
    a. ¿Qué categorías? Ovejas de cría ( ) Ovejas descarte ( ) Borregas 2 – 4 D ( ) Corderas ( ) Corderos ( ) Borregos ( ) Capones ( ) Carneros ( )
    b. ¿Con que frecuencia?

23. ¿Se realiza cuarentena de los animales antes de introducirlos al establecimiento? SI ( ) NO ( )
    a. Tiempo: menos 15 días ( ) 15-30 días ( ) 30-45 días ( ) 46-60 días ( ) 61 o más ( ) No se realiza ( )

Cuestión/ Comentario	Muy en desacuerdo	En desacuerdo	Ni de acuerdo ni en desacuerdo	De acuerdo	Totalmente de acuerdo
b. Los procedimientos de cuarentena son importantes para prevenir enfermedades					

24. ¿Existe un protocolo sanitario para animales que ingresan? SI ( ) NO ( )
    a. ¿Cuál?
25. Al introducir animales al predio, a. ¿se los inspecciona para constatar presencia de pietín? SI ( ) NO ( )

Cuestión/ Comentario	Muy en desacuerdo	En desacuerdo	Ni de acuerdo ni en desacuerdo	De acuerdo	Totalmente de acuerdo
b. Es importante observar las ovejas en busca de cojera antes de comprarlas					
c. Es importante examinar las patas de las ovejas antes de comprarlas					

26. ¿Se realiza tratamiento preventivo? SI ( ) NO ( )
    a. ¿Cuál?

27. ¿Posee potrero hospital estable? SI ( ) NO ( )
    a. Ubicación del mismo:

    b. ¿Qué animales son destinados a este potrero?

    c. ¿Considera que es seguro?
28. ¿Realiza descanso de los bretes cuando pasan estos animales? SI (   ) NO (   )
    a. Tiempo:
29. ¿Utiliza vacuna contra pietín? SI (   ) NO (   )
    a. Nombre comercial:
    b. ¿En qué categoría la utilizó?
    c. ¿Cuándo fue la última vez que la utilizó?
    d. ¿Vio efectos positivos en la majada? SI (   ) NO (   )
    e. ¿Volvería a usarla? SI (   ) NO (   )

Control de pietín:

30. ¿Hace cuánto observa el problema? __________ o (   ) No aplicable
31. ¿Quién realizó el diagnóstico de la enfermedad?
32. ¿Hay personal en el establecimiento que reconoce lesiones de pietín y sus grados? SI (   ) NO (   )
33. ¿Realizó alguna vez control y erradicación de la enfermedad? SI (   ) NO (   ).
    a. ¿Hace cuánto tiempo?
    b. ¿Fue un veterinario el responsable de la actividad? SI (   ) NO (   )
34. ¿Sienta los animales en algún momento del año para el control de afecciones podales? SI (   ) NO (   )
    a. Momento del año:
35. ¿Realiza despezuñado de los animales? SI (   ) NO (   ).
    a. Momento del año:
36. Pediluvio: SI (   ) NO (   )
    a. ¿En qué momento lo utiliza?
    b. ¿Qué utiliza para bañar a los animales?
    c. ¿Por cuánto tiempo deja los animales en el baño?
    d. ¿Cuándo fue el último baño realizado?
    e. ¿Cuántas ovejas adultas con medio vellón entran en el baño?
    f. Cuando lo utiliza, ¿tiene algún orden específico para manejar los animales?
    g. ¿Considera que el mismo es apto para el número de animales que se manejan en el establecimiento?
    h. ¿Obtiene los resultados esperados utilizándolo?
    i. Ubicación de mismo:
    j. Condiciones del mismo: Adecuada (   )   Inadecuada (   )

37. ¿Utiliza antibióticos para tratar animales enfermos? SI (   ) NO (   ).
    a. ¿Cuál?
    b. ¿En cuales casos? Crónicos o irrecuperables (   ) Recuperables (   )
38. ¿Elimina animales con pietín? SI (   ) NO (   )
    a. ¿Cuáles?
    b. ¿Qué hace con ellos? Embarque a frigoríficos (   ) Feria (   ) Consumo (   ) Sacrificio (   )
39. Complicaciones de la enfermedad:
    a. Miasis SI (   ) NO (   ) Si sí: 5-50% (   ) Más de 50% (   )
    b. Absceso podal SI (   ) NO (   ) Si sí: 5-50% (   ) Más de 50% (   )
    c. Dermatofilosis SI (   ) NO (   ) Si sí: 5-50% (   ) Más de 50% (   )
    d. Mortandad SI (   ) NO (   ) Si sí: 5-50% (   ) Más de 50% (   )
    e. Otros:
40. Opinión del productor/ capataz (respondiente)

Cuestión/ Comentario	Muy en desacuerdo	En desacuerdo	Ni de acuerdo ni en desacuerdo	De acuerdo	Totalmente de acuerdo
a. Considero un problema **menor** en la propiedad					
b. Considero un problema **importante** en la propiedad					
c. Considero una causa **importante** de pérdida económica					
d. La erradicación es una **prioridad**					
e. Pietín es **difícil** de erradicar					

Cuestión/ Comentario	Muy en desacuerdo	En desacuerdo	Ni de acuerdo ni en desacuerdo	De acuerdo	Totalmente de acuerdo
f. Siento **frustración** tratando de erradicarlo					
g. La anticipación de un brote me causa **estrés**					
h. Los brotes son **impredecibles**					
i. Hablo sobre el manejo de pietín con **mis vecinos**					

Nombre del evaluador: ______________________
