# Supplementary material for: Factors associated with ovine footrot lesions in Uruguayan flocks: a cross-sectional study
Source: Front Vet Sci. 2025 May 27;12:1585564. doi: 10.3389/fvets.2025.1585564 (PMC12148904; doi:10.3389/fvets.2025.1585564)
Supplement: Supplementary file 2 [file Data_Sheet_2.PDF]

Epidemiological study of Footrot in Uruguay

Date: _________

Property name: ____

Owner: ____

Department: ____

Location of the property:

____

DICOSE (unique and unrepeatable registration number in the Ministry of Livestock and Agriculture): ____

Property is: CASE ( ) CONTROL ( )

General characteristics of the property:

1. Total area: Hectares dedicated to sheep:
2. Soil type:
3. Sheep grazing: Natural pasture ( ) Improved pasture ( )
    a. Which improvements are used:
    b. Number of hectares:
4. Condition of perimeter fences: Good ( ) Fair ( ) Poor ( )
5. Approximate annual rainfall (mm):
6. a. How many people work on the farm? ___________ b. How many people are dedicated to sheep raising? _____________ c. How long have you been raising sheep? ________ d. Is there a high turnover of staff working with sheep? Quantify (e.g., average changeover time)
7. Breed:
8. a. Production objective: _____________________ b. Is it certified? Responsible Wool Standard ( ) Ecologic ( ) Other: __________
9. Number of sheep on the property
    a. Total stock:
    b. Breeding flock:
10. Do you raise cattle? YES ( ) NO ( )
11. Do you practice mixed grazing? YES ( ) NO ( )
12. When do you perform mating?
    a. Do you perform: ( ) Artificial insemination ( ) Natural mating ( ) Guided mating
13. When do you shear?

14. Do you supplement any category? YES ( ) NO ( )
    a. What category? Breeding ewes( ) Cull ewes( ) Ewes with 2 – 4 teeth( ) Ewe lambs ( ) Wether lambs ( ) Hoggets ( ) Wethers ( ) Rams ( )
    b. When do you supplement?

15. At any time during the year do you carry out enclosures in which the animals remain at high loading? YES ( ) NO ( )
    a. What category? Breeding ewes ( ) Cull ewes ( ) Ewes 2 – 4 teeth ( ) Ewe lambs ( ) Wether lambs ( ) Hoggets ( ) Wethers ( ) Rams ( )
    b. When?

16. Do you have dedicated veterinary assistance for sheep on the property? YES( ) NO ( )
    a. Annual ( ) Semi-annual ( ) Monthly ( ) Only upon request ( )
    b. Do you believe veterinary assistance is available to manage footrot in your region? YES ( ) NO ( )
    c. How easy is access to a veterinarian? Easy ( ) Moderate ( ) Difficult ( )

Biosecurity and sanitary conditions:

17. a. How many neighboring properties are there? ______ b. How many neighboring properties are there that produce sheep? ______
18. Do you take animals to large gatherings? YES ( ) NO ( ) If yes, where? Exhibitions ( ) Auctions ( ) Fairs ( )
19. Are the rams produced on the property or purchased from outside?
    a. If purchased from outside, do you ensure that the flock is free of footrot?
20. Do you use rams shared with other producers? YES ( ) NO ( )
    a. Did you observe any foot lesions on the animal(s)? YES ( ) NO ( )
21. a. Do you an immersion bath? YES ( ) NO ( )
    b. If yes, do you share it with neighbors? YES ( ) NO ( )
22. Do you bring animals onto the premises? YES ( ) NO ( )
    a. What categories? Breeding ewes ( ) Cull ewes ( ) Ewes 2 – 4 teeth ( ) Ewe lambs ( ) Wether lambs ( ) Hoggets ( ) Wethers ( ) Rams ( )
    b. How often?

23. Are animals quarantined before bringing them onto the premises? YES ( ) NO ( )

a. Time: less than 15 days( ) 15-30 days ( ) 30-45 days ( ) 46-60 days ( ) 61 days or more ( ) Not performed ( )

Question/ Comment	Strongly disagree	Disagree	Neither agree nor disagree	Agree	Strongly a gree
b. Quarantine procedures are important to prevent diseases					

24. Is there a health protocol for incoming animals? YES ( ) NO ( )
    a. Which one?
25. When animals are brought into the property, a. are they inspected for the presence of footrot? YES ( ) NO ( )

Question/ Comment	Strongly disagree	Disagree	Neither agree nor disagree	Agree	Strongly agree
b. It is important to check sheep for lameness before purchasing them					
c. It is important to examine sheep's feet before purchasing them					

26. Is preventive treatment performed? YES ( ) NO ( )
    a. Which one?

27. Do you have a stable hospital paddock? YES ( ) NO ( )
    a. Location:

    b. What animals are assigned to this paddock?

    c. Do you consider it safe?
28. Do you perform sanitary vacuuming of pens when these animals pass through? YES ( ) NO ( )

    a. How long:

29. Do you use footrot vaccines? YES (   ) NO (   )
    a. Trade name:
    b. In which category did you use it?
    c. When was the last time you used it?
    d. Did you see positive effects on the flocks? YES (   ) NO (   )
    e. Would you use it again? YES (   ) NO (   )

Footrot control:

30. How long have you observed the problem? __________ or (   ) Not applicable
31. Who diagnosed the disease?
32. Does the facility have staff who recognize footrot lesions and their stages? YES (   ) NO (   )
33. Have you ever carried out disease control and eradication? YES (   ) NO (   ).
    a. How long ago?
    b. Was the veterinarian responsible for the activity? YES (   ) NO (   )
34. Do you seat animals at any time during the year to control foot conditions? YES (   ) NO (   )
    a. Time of year:
35. Do you perform hoof trimming? YES (   ) NO (   ).
    a. Time of year:
36. Footbath: YES (   ) NO (   )
    a. When do you use it?
    b. What do you use to bathe animals?
    c. How long do you leave the animals in the bath?
    d. When was the last bath performed?
    e. How many adult sheep with half a fleece fit in the bath?
    f. When you use it, is there a specific order for handling the animals?
    g. Do you consider it suitable for the number of animals handled on the premises?
    h. Do you achieve the expected results?
    i. Location:
    j. Footbath condition: Adequate (   )   Inadequate (   )
37. Do you use antibiotics to treat sick animals? YES (   ) NO (   ).
    a. Which one?
    b. In which cases? Chronic or unrecoverable (   ) Recoverable (   )
38. Do you eliminate animals with footrot? YES (   ) NO (   )

a. Which ones?
b. What do you do with them? Shipping to slaughterhouses ( ) Fair ( ) Consumption ( ) Slaughter ( )

39. Complications of the disease:
    a. Myiasis YES ( ) NO ( ) If yes: 5-50% ( ) more than 50% ( )
    b. Foot abscess YES ( ) NO ( ) If yes: 5-50% ( ) More than 50% ( )
    c. Dermatophilosis YES ( ) NO ( ) If yes: 5-50% ( ) More than 50% ( )
    d. Mortality YES ( ) NO ( ) If yes: 5-50% ( ) More than 50% ( )
    e. Other:
40. Producer/ Foreman opinion (respondent)

Question/ Comment	Strongly disagree	Disagree	Neither agree nor disagree	Agree	Strongly agree
a. I consider it a minor problem on the property					
b. I consider it a major problem on the property					
c. I consider it a major cause of economic loss					
d. Eradication is a priority					
e. Footrot is difficult to eradicate					
f. I feel frustrated trying to eradicate it					
g. The anticipation of an outbreak causes me stress					
h. Outbreaks are unpredictable					
i. I talk about managing footrot with my neighbors					

Evaluator’s name: ________________________
